# Supplementary material for: Reproductive strategies in loggerhead sea turtle Caretta caretta: polyandry and polygyny in a Southwest Atlantic rookery
Source: PeerJ. 2025 Jan 7;13:e18714. doi: 10.7717/peerj.18714 (PMC11720971; doi:10.7717/peerj.18714)
Supplement: Supplemental Information 1 — Information from 43 loggerhead nests analyzed, including female body size (CCL, CCW, CA), the number of sampled and genotyped hatchlings, the estimated number of contributing males, and the presence of multiple paternity (MP). [file peerj-13-18714-s001.pdf]

Table S1. Information from the 43 loggerhead nests analyzed in this study, including the number of sampled hatchlings, the number of genotyped hatchlings, the estimated number of males contributing to each nest (N males) according to COLONY, and the presence or absence of MP. Bold represents females that nested in two seasons. *The final N males do not represent the total sum as there were males who contributed to more than one nest.

ID female/nest	Season	Sampled Hatchling	Genotyped Hatchlings	N males	MP?
SMV138	2017/18	36	15	1	No
SMV139	2017/18	25	6	2	Yes
SMV140	2017/18	27	19	1	No
**SMV141**	2017/18	40	15	3	Yes
SMV144	2017/18	25	8	6	Yes
SMV145	2017/18	23	7	3	Yes
SMV146	2017/18	35	14	1	No
SMV147	2017/18	22	5	3	Yes
SMV154	2017/18	29	7	3	Yes
SMV155	2017/18	23	7	3	Yes
SMV157	2017/18	22	5	2	Yes
SMV159	2017/18	28	7	3	Yes
SMV160	2017/18	37	12	3	Yes
SMV161	2017/18	26	12	3	Yes
SMV164	2017/18	66	18	1	No
SMV165	2017/18	32	12	5	Yes
SMV167	2017/18	33	15	2	Yes
SMV189	2018/19	67	17	2	Yes
SMV206	2018/19	21	5	2	Yes
SMV209	2018/19	18	15	3	Yes
SMV216	2018/19	25	20	3	Yes
SMV217	2018/19	20	20	1	No
SMV218	2018/19	22	17	1	No
SMV220	2018/19	30	20	2	Yes
SMV221	2018/19	25	18	2	Yes
SMV222	2018/19	7	7	2	Yes
SMV224	2018/19	38	18	3	Yes
SMV228	2018/19	33	7	6	Yes
SMV743	2019/20	23	20	1	No
**SMV141**	2019/20	30	18	4	Yes
SMV690	2019/20	20	7	2	Yes
SMV692	2019/20	20	12	4	Yes
SMV696	2019/20	9	6	5	Yes
SMV697	2019/20	20	18	1	No
SMV699	2019/20	20	18	1	No
SMV701	2019/20	8	5	3	Yes
SMV702	2019/20	13	3	3	Yes
SMV706	2019/20	8	5	1	No
SMV707	2019/20	23	16	1	No
SMV711	2019/20	11	5	2	Yes
SMV728	2019/20	20	13	2	Yes
SMV737	2019/20	29	20	1	No
SMV742	2019/20	20	20	2	Yes
**Total**		**1109**	**534**	**88***	**72.09%**
