# Supplementary material for: Reproductive strategies in loggerhead sea turtle Caretta caretta: polyandry and polygyny in a Southwest Atlantic rookery
Source: PeerJ. 2025 Jan 7;13:e18714. doi: 10.7717/peerj.18714 (PMC11720971; doi:10.7717/peerj.18714)
Supplement: Supplemental Information 2 — Details of loggerhead turtle males contributing to more than one nest within or between breeding seasons at Povoação Beach, Brazil, highlighting males exhibiting polygyny. [file peerj-13-18714-s002.pdf]

Table S2. Information from loggerhead turtle males that contributed to more than one nest within or between the three breeding seasons sampled. Underlined indicates the seven males that exhibited polygyny.

ID Male	Season	ID Female/Nest
*♂1	2017/18	SMV138
	2017/18	SMV165
	2017/18	SMV167
*♂3	2017/18	SMV139
	2017/18	SMV155
	2019/20	SMV696
*♂7	2017/18	SMV141
	2019/20	SMV692
*♂8	2017/18	SMV144
	2017/18	SMV147
	2019/20	SMV692
*♂11	2017/18	SMV144
	2019/20	SMV692
*♂17	2017/18	SMV146
	2018/19	SMV228
*♂21	2017/18	SMV154
	2017/18	SMV159
	2019/20	SMV141
*♂22	2017/18	SMV154
	2017/18	SMV160
*♂25	2017/18	SMV157
	2018/19	SMV216
	2018/19	SMV220
*♂28	2017/18	SMV159
	2017/18	SMV160
*♂35	2017/18	SMV165
	2019/20	SMV706
*♂41	2018/19	SMV206
	2019/20	SMV702
