# Supplementary material for: Reproductive strategies in loggerhead sea turtle Caretta caretta: polyandry and polygyny in a Southwest Atlantic rookery
Source: PeerJ. 2025 Jan 7;13:e18714. doi: 10.7717/peerj.18714 (PMC11720971; doi:10.7717/peerj.18714)
Supplement: Supplemental Information 3 — Literature review of multiple paternity rates in wild loggerhead sea turtle clutches, including maximum number of sires, average number of fathers per nest, and the genetic techniques used for analysis. [file peerj-13-18714-s003.pdf]

Table S3. Compiled literature on multiple paternity in wild loggerhead sea turtle clutches.

**Regions**	**Clutches with multiple paternity, in percentage (number of clutches)**	**Max number of sires**	**Average number of fathers per nest**	**Technique used (number of loci)**	**Referen ce**
Queensland (Australia)	33%? (45)	2	?	Allozymes (13)	Harry & Briscoe, 1988
Florida (USA)	31% (70)	3	1.40	Microsatellites (4)	Moore & Ball, 2002
Florida (USA)	22% (36)	5	1.56	Microsatellites (7)	Lasala et al., 2020
Florida (USA)	33% (3)	3	1.67	Microsatellites (2)	Bollmer et al., 1999
Queensland (Australia)	65.5% (29)	5	1.79	Microsatellites (6)	Howe et al., 2018
Western Australia (Australia)	48% (25)	2	1.80	Microsatellites (4)	Tedeschi et al., 2015
**Espírito Santo (Brazil)**	**72.09% (43)**	**6**	**2.04**	**Microsatellites (4)**	**This study**
Dalyan Beach (Turkey)	70% (25)	3	2.08	Microsatellites (2)	Sari et al., 2017
Florida (USA)	70% (51)	7	2.47	Microsatellites (7)	Lasala et al., 2018
Georgia (USA)	78% (72)	7	2.72	Microsatellites (5)	Lasala et al., 2013
Zakynthos (Greece)	95% (21)	5	3.50	Microsatellites (4)	Zbinden et al., 2007
